# Bereavement care practices: A national survey of Australian paediatric intensive care units

**DOI:** 10.1016/j.ccrj.2026.100221

**Published:** 2026-09-23

**Authors:** Arielle N. Jolly, Kristen S. Gibbons, Ashleigh E. Butler, Simon Erickson, Kylie Davies, Kate Masterson, Jason M. Sharbanee, Fenella J. Gill, the ANZICS Paediatric Study Group

**Affiliations:** a School of Nursing, Curtin University, Bentley, Western Australia, Australia; b Paediatric Critical Care, Child and Adolescent Health Service, Nedlands, Western Australia, Australia; c Children's Intensive Care Research Program, Child Health Research Centre, Faculty of Health, Medicine, and Behavioural Sciences, The University of Queensland, Brisbane, Queensland, Australia; d School of Nursing and Midwifery, La Trobe University, Australia; e Paediatric Intensive Care Research, Royal Children's Hospital, Melbourne, Victoria, Australia; f enAble Institute, School of Public Health, Curtin University, Bentley, Western Australia, Australia; g Nursing Research, Child and Adolescent Health Service, Nedlands, Western Australia, Australia

**Keywords:** Intensive care units, Paediatric, Australia, Critical care, Bereavement, Surveys and questionnaires

## Abstract

**Objectives:**

Hospital-based bereavement care is an important source of support for families following the death of a child in a paediatric intensive care unit (PICU) but has been identified as lacking in many critical care units worldwide. Recent surveys exploring bereavement practices in intensive care units have not reported comprehensive paediatric data, meaning the extent of this problem within the PICU setting is currently unknown. The aim of this study was to describe current bereavement care practices in Australia PICUs.

**Design:**

A cross-sectional, self-reported, online survey, conducted between November 2024 and April 2025. A validated 34-point critical care bereavement survey was adapted for the paediatric setting and assessed for face validity with five PICU nurses before circulation.

**Participants:**

Representatives from Australian PICUs were surveyed via the Australian and New Zealand Intensive Care Society Paediatric Study Group mailing list.

**Results:**

Completed surveys were received from 11 dedicated PICUs in Australia (100% response rate). Most respondents reported more than half of the 10 elements of bereavement care provided within their PICUs, but only two PICUs (2/11 [18%]) provided a formal bereavement follow-up service. Six PICUs (6/11 [55%]) provided an informal service, while a further three PICUs (3/11 [27%]) provided no follow-up service. Only one unit (1/11 [9%]) reported paid, dedicated time allocated to bereavement.

**Conclusions:**

This national survey showed bereavement care provided within Australian PICUs was similar across units, but formal bereavement follow-up services were lacking. Bereavement follow-up in most Australian PICUs was delivered by clinicians within their other roles or in unpaid time. We suggest further research into family bereavement needs to strengthen the evidence-base for PICU bereavement care, along with increased training, resourcing, and funding for PICU clinicians to deliver bereavement follow-up services.

## 1 Introduction

Most children admitted to a paediatric intensive care unit (PICU) in mid- to high-resource settings will survive their admission, benefitting from life-sustaining treatments provided by highly skilled clinicians, including doctors, nurses, and allied health professionals.[1], [2], [3] For the minority (2–9%) who do not survive, PICU clinicians pivot from a focus on survival to the provision of family-centred grief and bereavement care to support the child and their relatives before, during and after the death.^2^^,^^4^ For many families, bereavement experienced within the complex and technical environment of a PICU can come as a sudden shock, accompanied by feelings of alienation and trauma that may pose a threat to adaptive grief.[5], [6], [7], [8], [9] Potential adverse grief outcomes for these families represent low-frequency, high-impact PICU sequelae, with psychosocial,^10^ physical,^11^ relational,^12^ and financial impacts^13^ of grief affecting parents,^14^ siblings,^15^ extended family,^16^^,^^17^ and the communities they grieve in.^18^

The death of a child causes a devastating form of grief, one that can leave families feeling torn apart.^19^^,^^20^ Approximately 40% of parents of children who die in a PICU setting meet the diagnostic threshold for a post-traumatic stress disorder diagnosis,^21^ while as many as 60% demonstrate high levels of complicated grief that persists over time.^4^ Other potential adverse grief effects include depression, family dysfunction, and physical ill health.^10^^,^^16^^,^^22^ Risk stratification models place grief over the death of a child in the highest bereavement risk category,^23^ suggesting most, if not all, families bereaved in the PICU could benefit from professional intervention. Hospital-based bereavement care has been recognised as an important source of support for families who experience the death of a child in a PICU but has been identified as inaccessible and inadequate in many intensive care units (ICUs) worldwide.^24^

Several surveys have mapped the availability and scope of grief and bereavement care offered by ICUs across primarily high-income settings, including Australia, New Zealand, and Europe.[25], [26], [27], [28] These surveys have identified grief and bereavement care is generally delivered well before and during death within the ICU setting, including adult ICU, PICU, and neonatal intensive care units, but formal services for delivery of bereavement follow-up after death are lacking.[25], [26], [27], [28] While previous surveys provide an overview of current ICU bereavement practices, they have not focused specifically on the PICU setting or reported comprehensive paediatric data.[25], [26], [27], [28] The minimal paediatric data included means the extent of this problem within PICU settings remains unknown.

### 1.1 Aim

The aim of this national survey was to understand current bereavement care practices in Australian PICUs.

## 2 Methods

A cross-sectional, self-reported, online survey was conducted between November 2024 and April 2025 to explore bereavement practices in Australian PICUs and identify gaps in care and is reported according to the Checklist for Reporting Results of Internet E-surveys.^30^

### 2.1 Survey instrument

The survey for this study was adapted and used with permission from Butler et al.^21^ and Egerod et al.,^16^ based on an original, validated 34-point ICU bereavement survey developed by Mitchell et al.^19^ The original survey demonstrated content validity through a two-round review process involving a total of 18 clinicians, academics, and researchers from both Australia and New Zealand.^27^ Minor contextual adaptations were made for the paediatric setting by adding the word “paediatric” before each instance of the phrase “intensive care” within the survey, and substituting “PICU” where the acronym “ICU” appeared. Three paediatric options were added to a list of bereavement care elements in Q9 (See Supp Item 1), including memory-making, sibling bereavement resources, and end-of-life photography. Face validity testing of the modifications was undertaken with five PICU nurses, resulting in minor refinements to the punctuation.^31^ A web-based survey was developed for this study from the finalised survey questions using secure Research Electronic Data Capture (REDCap; Vanderbilt, Nashville) tools hosted at the Western Australian Child and Adolescent Service.^32^^,^^33^ The open voluntary survey was accessible via an email link, and all 34 items appeared on one page, with no review step included.

### 2.2 Sample and recruitment

All PICUs in Australia were eligible to participate, while adult ICUs, neonatal intensive care units, and mixed ICUs were excluded. Careful consideration was given to including New Zealand; however, a separate ethical approval was required to survey the single dedicated PICU located in New Zealand, which already has a formal, established, funded PICU bereavement service. As the details of this service were publicly available online, the status of PICU bereavement care in New Zealand was considered already known. An email invitation was circulated to Australian PICU clinicians via the official mailing list of Australian and New Zealand Intensive Care Society Paediatric Study Group (ANZICS PSG), which had endorsed the study. This contained information about the purpose of the study, the target respondents, the voluntary nature of the study, and helpline contact details in case of distress while completing the survey. Respondents were given the option to provide their email address and were informed that data would be stored securely and that no identifying data would be reported.

Target respondents were PICU research coordinators, who were requested to complete a single survey per site on behalf of the PICU in which they were employed. Research coordinators were selected due their investment in this study through collaboration with the ANZICS PSG and their professional skillset. In Australia, PICU research coordinators are typically nurses with clinical PICU experience, who work closely with their multidisciplinary PICU team to conduct clinical research projects, many of which are endorsed by the ANZICS PSG.

For this survey, collaboration steps included presenting the study at an ANZICS PSG virtual scientific conference prior to survey circulation, where attendees were primarily PICU doctors and research coordinators. Feedback on the survey content, inclusion and exclusion criteria, and survey circulation plan were received from the group and informed the final methodology. Research coordinators agreed that by nature, their role involved gathering data from diverse clinical sources; therefore, they were well placed to collate information about PICU bereavement practices from their colleagues, including doctors, nurses, social workers, and other bereavement stakeholders, in order to answer the survey questions on behalf of their PICU. Respondents were not expected to represent the diverse views of clinicians but rather to report bereavement care and services available within their PICU. Survey instructions explicitly informed respondents to consult bereavement stakeholders in their organisation to ensure their responses accurately and fully reflected current practices.

### 2.3 Setting

In Australia, paediatric intensive care services are highly centralised and PICUs are primarily located in metropolitan tertiary paediatric hospitals, accepting referrals from and acting as paediatric critical care resource centres for other metropolitan, regional, and remote hospitals in their geographic area.^34^ Some PICUs are located in university-affiliated general hospitals.^34^ There are 11 dedicated PICUs across Australia,^35^ as well as additional paediatric beds/areas within adult ICUs, and a combined neonatal and paediatric ICU located in Tasmania.^34^

### 2.4 Data analysis

Data were exported from REDCap into Microsoft Excel (Microsoft Corporation, Washington) for descriptive analysis.^36^ Due to the small number of PICUs in Australia, results are presented as proportions and percentages only. Open-ended questions were analysed using inductive qualitative content analysis and managed in Excel.^37^ Iterative coding was performed through examining common issues raised in the data, which were highlighted, grouped, and categorised by primary author AJ, and reviewed by AEB, FG, and KG. Simple counts were used to quantify the proportion of comments relating to each category and are presented alongside descriptive data.

### 2.5 Ethical approval

Ethical approval for this study was granted by the Human Research Ethics Committees of the Child and Adolescent Health Service (RGS6539) and Curtin University (HRE2024-0121).

## 3 Results

### 3.1 Personal and unit demographics

Eleven valid responses were received, representing a 100% response rate of one survey from each dedicated Australian PICU. Respondents included their email address which enabled verification that a complete response was received from each unit (see Fig. 1). Four incomplete responses were received and two duplications, which were deemed invalid and not included in the final analysis. As presented in Table 1, most PICUs were located in metropolitan locations (9/11 [82%]), on the Eastern Coast of Australia (10/11 [91%]) where patients from a range of clinical specialties were cared for—most commonly general paediatrics (11/11 [100%]), neurology/neurosurgery (9/11 [82%]), and cardiothoracics/cardiology (8/11 [73%]). Some respondents added additional specialties in free text, including oncology (4/11 [36%]); trauma (4/11 [36%]); transplants (2/11 [18%]); and ear, nose, and throat (1/11 [9%]).

**Table 1:** Demographic details (N = 11).

		n
Paediatric intensive care unit type	Public hospital	10
	Private hospital	1
	Metropolitan setting	9
	Regional setting	2
Australian state or territory	Australian Capital Territory	0
	New South Wales	3
	Queensland	4
	South Australia	1
	Tasmania	0
	Victoria	2
	Western Australia	1
Survey respondent’s role	Research coordinator	7
	Nurse unit manager	1
	Medical staff specialist	1
	Social worker	1
	Clinical nurse consultant	1

**Fig. 1:**
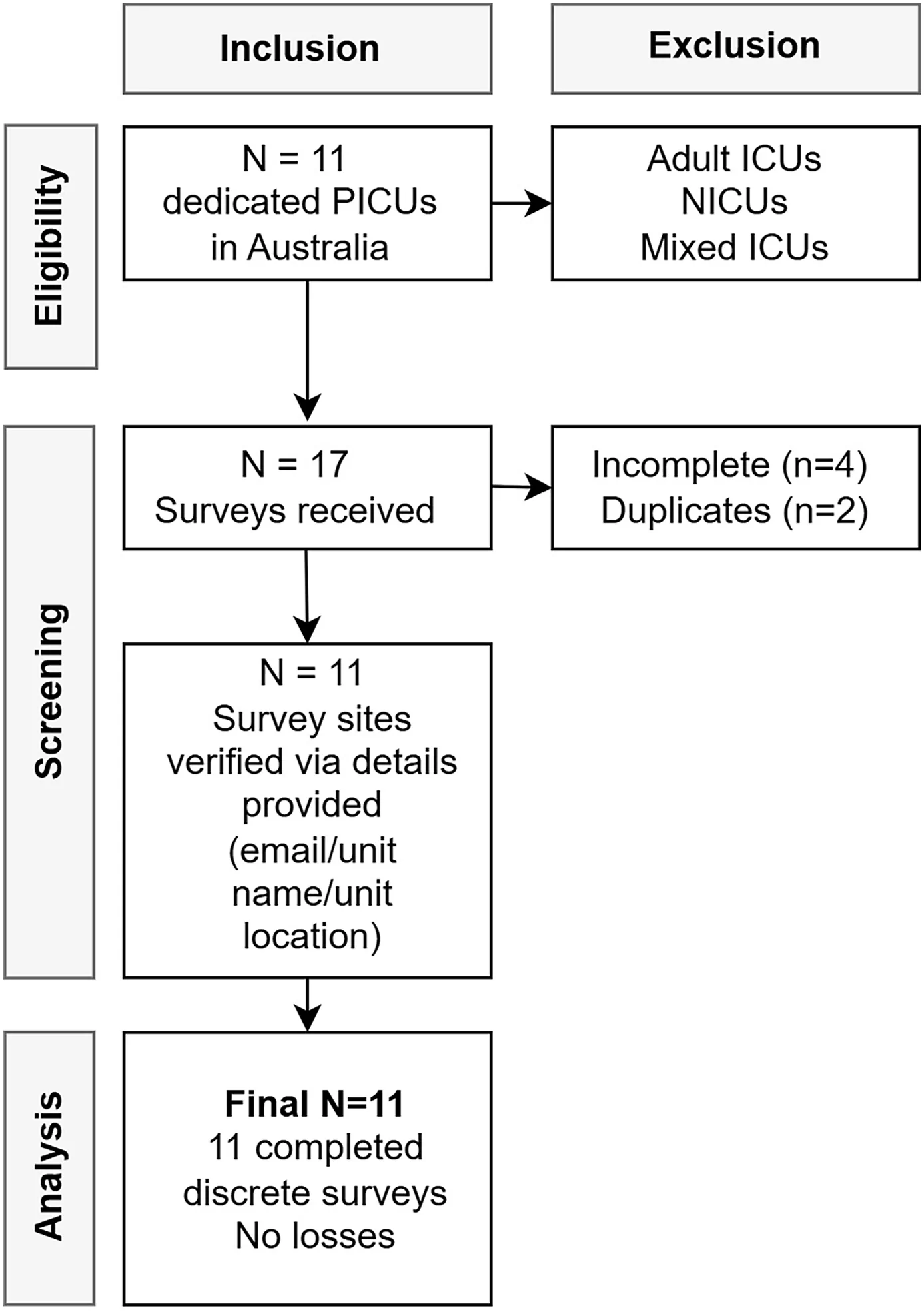
Survey response flow chart. ICU: intensive care unit; NICU: neonatal intensive care unit; PICU: paediatric intensive care unit.

Two PICUs were located in regional areas (2/11 [18%]), and there were no responses from the Northern Territory, Australian Capital Territory, or Tasmania where no dedicated PICUs are located. Survey respondents were primarily research coordinators (7/11 [64%]), with one response each from a nurse unit manager, medical staff specialist, social worker, and clinical nurse consultant. These responses were from smaller units with minimal research coordinator hours. Respondents were experienced clinicians, most with more than 10 years of clinical experience, and greater than five years PICU experience (10/11 [91%]).

### 3.2 General bereavement care elements

The most common PICU bereavement care elements reported were memory making (11/11 [100%]) and end-of-life photography (11/11 [100%]), followed by sibling involvement (10/11 [91%]), bathing and dressing the deceased (10/11 [91%]), viewing in the PICU (10/11 [91%]), and viewing in the hospital mortuary (10/11 [91%]). Most bereavement care practices were reported within most units. Three units (3/11 27%]) reported all 10 bereavement care elements listed as multiple-choice options (Fig. 2), while two also added an additional free-text bereavement care item, an annual memorial (2/11 [18%]), and one listed the use of a bereavement suite.

**Fig. 2:**
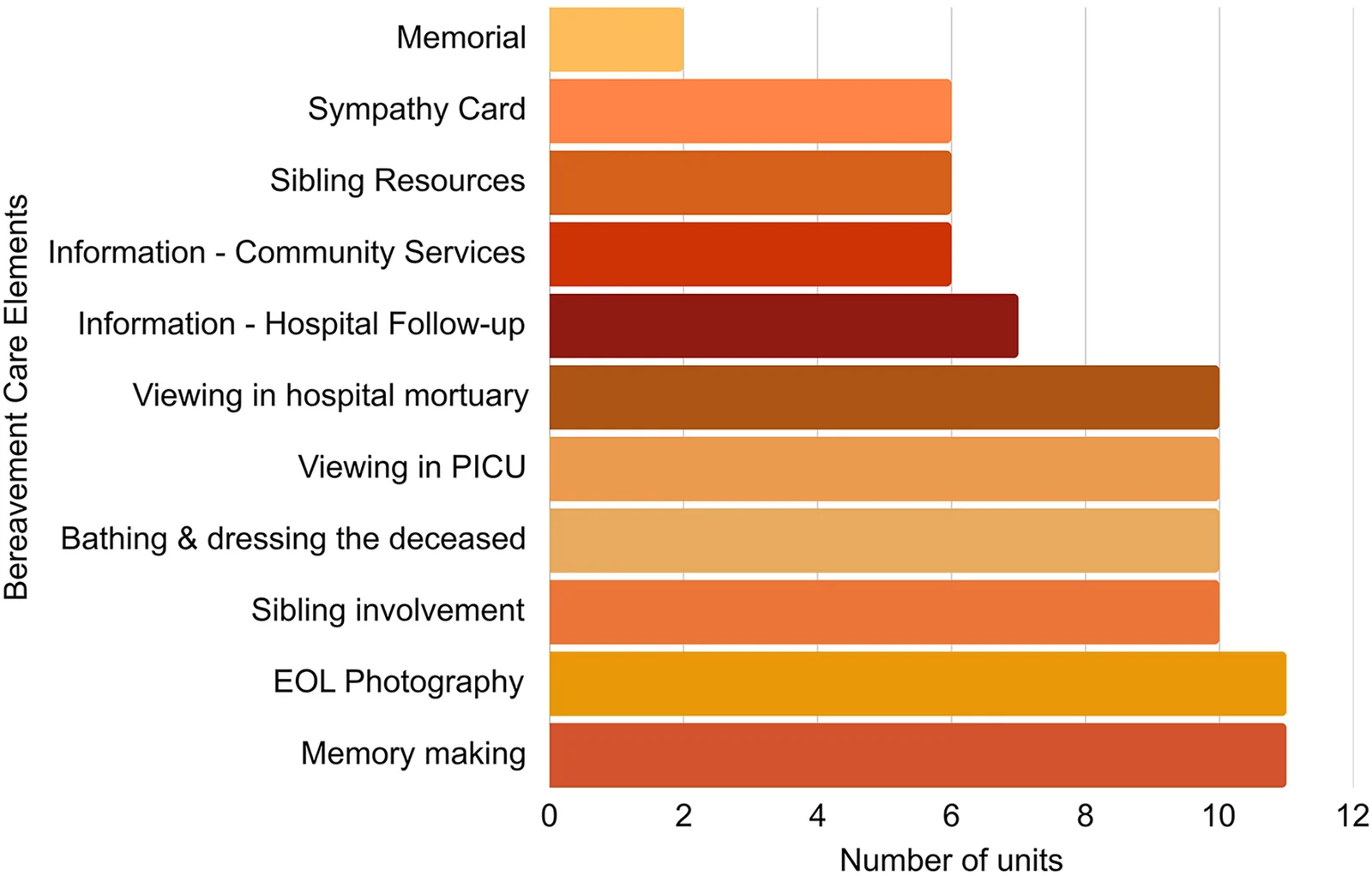
Bereavement care elements offered in respondent PICUs (N = 11). EOL: end of life; PICU: paediatric intensive care unit.

### 3.3 Model of bereavement services

Of the 11 survey respondents, two reported a formal bereavement follow-up service (2/11 [18%]) and six reported an informal service (6/11 [55%]), while three units reported no follow-up service (3/11 [27%]). The units where formal bereavement follow-up services were reported had these established for 5–15 years.

Across the eight units where a bereavement service was reported, both formal and informal, all (8/8 [100%]) included a telephone call to families after the child’s death. These calls were primarily carried out by a social worker (3/8 [38%]) or a doctor (2/8 [25%]) or according to family request. Most services, whether formal or informal, included the offer of a return visit to the PICU after the child’s death (5/8 [63%]), but the timing and visit details varied between units. Half included formal counselling for families as a component of their service, and all eight included a medical bereavement meeting (Table 2). The timing of medical bereavement meetings ranged from 1 to 6 weeks after the child’s death and were in equal numbers offered routinely (4/8 [50%]) or at the request of families (4/8 [50%]). In three units, bereavement meetings were conducted at a location of the family’s choice (3/8 [38%]), while in a further three, meetings were held in the PICU (3/8 [38%]), and for the remaining two, the location was not specified.

**Table 2:** Bereavement follow-up service elements (N = 8).

		n
Type of service	Formal	2
	Informal	6
No of years since establishment	5–10 years	1
	10–15 years	1
	Unknown	6
**Care elements offered**		
Telephone call	Yes	8
	No	0
PICU visit	Yes	5
	No	3
Formal counselling	Yes	4
	No	4
Bereavement meeting	Yes	8
	No	0

PICU: paediatric intensive care unit.

Various additional supports were reported by respondents from units where bereavement services were offered. These included referral to follow-up from other teams, including sepsis-specific services (1/8), referral to bereavement services offered by a larger, tertiary paediatric hospital in the same state (1/8), and links to community grief resources and services (3/8). Additionally, one respondent from a PICU without a bereavement service reported in free comments families could be referred to a larger, tertiary paediatric hospital in the same state for bereavement follow-up.

### 3.4 Bereavement service workforce

The staffing profile of bereavement services, both formal and informal, are shown in Table 3. Services were managed primarily by social workers (7/8 [88%]) and/or nursing unit managers (2/8 [25%]), while follow-up care was predominantly provided by a social worker (7/8 [88%]) in some units (2/8 [25%]) accompanied by another professional such as a psychologist or pastoral carer. Two to three staff members were involved in providing bereavement follow-up in most units (5/8 [63%]). One reported staff holding permanent occupancy in a bereavement role (1/8 [13%]), while the majority reported bereavement care was incorporated into other duties (6/8 [75%]), including social work, nurse manager, and care coordination roles. Funding for bereavement services varied across units. Only one unit reported dedicated, paid time for bereavement follow-up (1/8 [13%]). Four of the six informal bereavement services involved unpaid, voluntary time for staff to complete bereavement duties (4/6 [67%]).

**Table 3:** Bereavement follow-up service staffing profile (N = 8) and organisational support (N = 11).

Bereavement follow-up staffing profile (N = 8)		n
Service management	Social worker	6
	Nurse unit manager	2
Service delivery	Social worker	7
	PICU consultant	2
	Psychologist or counsellor	2
	Pastoral carer	2
Number of staff involved	1 staff member	3
	2–3 staff members	5
Hours allocated	No specific hours allocated	7
	Varies according to need	1
Staff occupancy	Permanent role	1
	Rotating role	1
	Duties incorporated into another role	6
Funding	Dedicated, paid time	1
	Funded time, not allocated to follow-up	2
	Voluntary service	4
	Unsure	1
**Support for bereavement care (N = 11)**		**n**

Debriefs provided	Yes, routinely	6
	Yes, on request	3
	Unsure	2
Type of debrief	Both hot and cold	8
	Cold only	1
	Unsure	2
Bereavement training	Yes, self-directed	2
	Yes, external to the PICU	4
	No	3
	Unsure	2

PICU: paediatric intensive care unit.

Organisational support for bereavement care varied unit to unit and was reported by all respondents, whether or not their PICU had a bereavement follow-up service. Of the 11 respondents, three (3/11 [27%]) reported no extra support or training for bereavement work. Of those who did report extra training, some commented that it was self-directed and ad hoc (2/11 [18%]) or provided externally by palliative care specialists rather than internally within the PICU (4/11 [36%]). In most units, staff members were supported with debriefs after patient deaths (9/11 [82%]), most of these routinely (6/11 [55%]) and the remainder on request.

### 3.5 Service evaluation

Service evaluation was limited. One unit reported formal service evaluation, and one informal service was evaluated via word-of-mouth. From these evaluations and free comments from respondents where their service had not been evaluated, respondents indicated the components of bereavement follow-up most appreciated by families were bereavement meetings (2/11 [18%]), and individual supports including phone calls and ongoing connection (2/11 [18%]). Least appreciated components were when the person who called did not know the family and their individual story (1/11 [9%]). Due to few reported evaluations across units, views on acceptability of current services to families were limited to these comments.

Feedback from clinicians completing the survey was included in free-text comments at the conclusion. Comments centred around inconsistency (5/11 [45%]), with bereavement support described as “ad hoc” (2/11 [18%]) or more positively as “individualised” to the needs of different families (1/11 [9%]), and the importance of “designing a service that is equitable to all bereaved families” was noted (1/11 [9%]). Tension between clinician and management priorities was highlighted by three respondents (3/11 [27%]), who reported “care coordinators tried to get a standard service set up [but] value was not seen by management”, an existing schedule of bereavement phone calls was “felt by staff not to be enough, and staff often make additional calls”, and “staff question why there is no funding for bereavement follow-up”.

## 4 Discussion

This national survey was the first to comprehensively explore bereavement practices in Australian PICUs, reporting responses from all 11 dedicated PICUs across the nation. This small sample size represents the entire population of dedicated PICUs under investigation. The central finding was while most bereavement care elements for dying children and their families were available within Australian PICUs, few had formal, funded bereavement follow-up services to support families afterwards. In Australian PICUs, bereavement follow-up was mostly delivered as informal, unfunded, and voluntarily care provided by staff within other clinical roles.

Previous studies into ICU bereavement care have not explored paediatric practices exhaustively. A 2021 multicentre survey study focused on international PICU bereavement practices but did not report follow-up care for families after death.^24^ The Pediatric Intensive Care Unit Model of Integrated Care study included 34 participating PICUs from 18 countries across Asia, Latin America, North America, Europe, and Africa, surveying unit infrastructure and adherence to palliative care and grief and bereavement guidelines.^24^ Across all study centres, fulfilment of guidelines reached an average score of 37.5%, with availability of grief and bereavement resources higher among units in resource-rich countries than in lower-income countries.^24^ To date, the Pediatric Intensive Care Unit Model of Integrated Care study reported the most comprehensive data on PICU grief and bereavement care but did not illuminate PICU bereavement follow-up practices internationally.

The original survey adapted for the current study was utilised in a 2015 study of bereavement practices across primarily adult ICUs in Australian and New Zealand. The 2015 survey, which included eight PICUs (5% of total responses), found less than a third of all respondent ICUs in both countries offered formal bereavement follow-up services.^27^^,^^28^ A follow-up survey of 125 Australia and New Zealand ICUs conducted in 2023, including five PICUs (5%), found only 36 ICUs (29%) offered formal bereavement follow-up services, representing just two additional sites since the 2015 survey.^29^ A 2019 European study using the same instrument surveyed 85 critical care nurses from 18 primarily Scandinavian countries, including three PICU respondents (4%), finding that 35 of 85 respondents (41%) reported a bereavement follow-up service provided by their ICU.^26^ These primarily adult surveys all reported a minority of ICUs with formal, funded bereavement follow-up services for families.

With PICU as the sole focus of this study, key contrasts to bereavement practices seen in predominantly adult ICU settings were identified.^26^^,^^27^^,^^29^ Importantly, two of the three paediatric specific bereavement elements added to this survey, memory-making and end-of-life photography, were rated as the most commonly delivered aspects of bereavement care in Australian PICUs, compared to viewing the deceased in the ICU as the most common bereavement care reported in adult ICU surveys.^26^^,^^27^^,^^29^ These findings suggest bereavement care is performed differently in paediatric settings to adult settings, highlighting the principle that paediatric intensive care is a specialty with unique practices.^38^ What was not shown by the clinician-reported findings is whether current PICU practices meet the needs of grieving families. The distinctive challenges of bereavement in the PICU, and the complexity of meeting family needs have been identified in other research,^39^ with Meert et al.^40^ describing the PICU as “a special context for parental bereavement” (p.712). We suggest a focus on paediatric-specific priorities in future critical care bereavement research to strengthen the evidence base for care and services to meet the needs of children and their families at end of life and beyond.

While our findings showed similarities across bereavement care elements offered within Australian PICUs, inconsistency was seen in the availability, resourcing, and staffing of bereavement follow-up services. Just over 70% of PICUs (8/11) offered bereavement follow-up of some kind, although only 18% reported formal services. This contrasts with the 30–40% of units offering formal follow-up services in predominantly adult ICU settings.^27^^,^^29^ While our results indicated most Australian PICUs offered bereavement follow-up, only a small minority of these services were formal, funded, and permanently staffed. This underscores structural challenges to the delivery and maintenance of high-quality bereavement follow-up services, also reported in the wider ICU context.^24^^,^^26^^,^^27^^,^^29^ In the paediatric setting of this study, follow-up was provided despite these challenges, with clinicians carrying out the work of family follow-up support voluntarily or in time allocated to other clinical tasks. We suggest PICU clinicians may value provision of bereavement follow-up enough to dedicate time regardless of organisational support; a view supported by research identifying transitional hospital bereavement care as essential for families after a child’s death.^41^ However, reliance on clinicians providing bereavement follow-up because they care about it means that service delivery effectively ends when those dedicated staff members leave or become overwhelmed, which threatens the sustainability of service delivery and family experience. Conversely, funded, resourced services in health care typically also rely on dedicated, passionate clinician champions to succeed over time.^42^ Research into clinician perspectives on PICU bereavement follow-up is lacking, and further inquiry could potentially illuminate these issues.

Another key finding was the inconsistency of bereavement training provided for clinicians in Australian PICUs. While training was available at most sites, clinicians reported seeking it for themselves, and training was not usually available within the PICU but delivered by palliative care teams. These findings echo past research which has highlighted a need for specific grief, loss, and bereavement training for clinicians, relevant to their clinical specialty.^41^ Palliative care specialists are highly skilled in the delivery of bereavement care and well placed to deliver training; however, critical care settings have distinctive complexities indicating a need for additional specialty-specific training. To ensure PICU clinicians are well equipped to provide end-of-life, grief, and bereavement care in all the critical scenarios they may encounter, readily accessible training delivered from a palliative and critical care lens could provide an important foundation for skill development, alongside clinical experience and mentorship.^43^ Several simulation-based training modules have been developed to support critical care clinicians to manage difficult conversations,^44^^,^^45^ organ donation scenarios,^46^ family presence during cardiopulmonary resuscitation,^47^ and postevent debriefing.^45^^,^^48^ A practical structure for comprehensive PICU grief and bereavement training might involve utilising, tailoring, and extending these existing approaches.^45^^,^[49], [50], [51] Recommendations from literature also suggest integrating grief theory,^51^^,^^52^ cultural competency,^49^^,^^51^ therapeutic communication,^53^ end-of-life ethical considerations,^51^ and guidance on follow-up techniques into bereavement training.^54^

In addition to structural challenges to the provision of formal PICU bereavement follow-up, we also found most bereavement services had not been evaluated. Other studies have similarly identified a lack of formal evaluation of ICU bereavement follow-up services.^26^^,^^27^^,^^29^ Without evaluation, the quality of services provided and acceptability to families remain unknown.^55^ With little research reporting the perspectives of bereaved families in the PICU setting, the evidence base guiding PICU bereavement follow-up practices remains uncertain.^55^ To improve evaluation of bereavement care practices in the PICU setting, the Bereaved Parent Needs Assessment tool was developed and validated in 2012, providing an evidence-based framework against which to assess existing practices.^39^^,^^56^ Current recommendations from the literature suggest PICU bereavement services should involve transitional support from hospital to home,^57^ including resources to support family grief needs,^57^ guidance for families engaging with the coroner,^57^ a postbereavement meeting or debrief,^58^ access to counselling and referral to specialist grief services in the community,^59^ ongoing contact with clinicians familiar to the family,^60^ follow-up for the whole family, rather than parents alone,^60^ acknowledgement of anniversaries and special dates,^60^ and scheduled contact points such as 1 week post bereavement, 3 months, and 1 year.^60^ These recommendations provide a practical foundation for bereavement care; however, further research is indicated to strengthen the evidence base and ensure that PICU practices reflect family-centred principles and real-world priorities.^24^^,^^41^^,^^55^

### 4.1 Strengths and limitations

This study had several strengths and limitations. The high response rate was a strength, as was the level of clinical experience amongst respondents, potentially increasing the reliability of survey responses. The minor contextual adaptation of a validated instrument supported the credibility of results and facilitated comparison with previous surveys completed in the ICU context. Limitations included the self-reported nature of the survey, meaning responses may not reflect all aspects of bereavement care practice within individual PICUs. Second, organisations may define “bereavement service” differently or have varying thresholds for classifying a service as “formal” or “informal”. Respondents were not asked how long families were supported by PICU bereavement follow-up, so the dose of follow-up provided by each service is not comparable across units. The survey used in this study contained no questions about religious or cultural bereavement practices, and therefore the results do not establish the acceptability of existing bereavement practices for indigenous, first nations, or culturally diverse groups. Furthermore, as the survey was completed by clinicians, the views of bereaved families about whether current practices adequately support their needs are not described. Finally, the survey did not contain any questions about guidelines or policies governing bereavement practices, meaning the evidence base for care offered within each PICU was not demonstrated by this study. This is an important limitation as guidelines and policies provide the foundation for standardisation of care, quality assessment, and ongoing service improvements, which are needed to ensure consistent, family-centred bereavement care. To assess policies and procedures guiding current practice, how they are operationalised, and whether their implementation is associated with improved family outcomes, further work is needed.

## 5 Conclusion

This study showed a similar array of bereavement care elements were provided to dying children and their families within most Australian PICUs; however, few formal bereavement services were in place to provide follow-up support afterwards. Bereavement follow-up was mostly provided by PICU clinicians without dedicated time for the role, and clinicians often had to seek self-directed bereavement training outside the PICU to support their practice. Furthermore, a minority of existing PICU bereavement follow-up services had been evaluated, meaning the quality of current support and acceptability to families who receive it remains largely unknown. More work needs to be done to explore family bereavement needs and experiences to develop the evidence-base for family-centred PICU bereavement follow-up services and to ensure such services are supported with comprehensive training, funding, and dedicated staffing.

## Data availability statement

Due to the small sample size and risk of identification of individual sites, participants were assured raw data would remain confidential and not be shared.

## Funding

This study was supported financially by grant funding from the Perth Children’s Hospital Foundation (PCHF10159), Stan Perron Charitable Foundation (00058ProgPart) and the Future’s Health and Research Innovation Fund (MRAS22-23/4). Arielle Jolly’s work on this study was supported via a Nursing Research Fellowship from the Child and Adolescent Health Service and the Telethon Trust. Fenella Gill’s research program is supported by the Stan Perron Charitable Foundation. Kristen Gibbons is supported by a National health and Medical Research Council Emerging Leader Fellowship. Ashleigh Butler is an Australian Research Council Discovery Early Career Researcher Award recipient for research into “Supporting the inclusion of siblings when a family is in crisis” (DE230101123). The funders had no part in the study design, conduct, or data analysis and did not have any authority over these activities.

## CRediT authorship contribution statement

Arielle Jolly: Conceptualisation, data curation, formal analysis, funding acquisition, investigation, methodology, project administration, resources, visualisation, writing – original draft. Kristen Gibbons: Conceptualisation, methodology, supervision, validation, visualisation, writing – review & editing. Ashleigh Butler: Conceptualisation, methodology, supervision, validation, visualisation, writing – review & editing. Simon Erickson: Funding acquisition, conceptualisation, methodology, supervision, validation, visualisation, writing – review & editing. Kylie Davies: Methodology, supervision, validation, visualisation, writing – review & editing. Kate Masterson: Conceptualisation, methodology, writing – review & editing. Jason Sharbanee – Supervision, writing – review & editing. Fenella Gill: Conceptualisation, methodology, supervision, data curation, formal analysis, validation, visualisation, writing – review & editing.

## Conflict of interest

The authors declare that they have no known competing financial interests or personal relationships that could have appeared to influence the work reported in this manuscript.
