# Supplementary material for: Bereavement care practices: A national survey of Australian paediatric intensive care units
Source: Crit Care Resusc. 2026 Sep 23;28(4):100221. doi: 10.1016/j.ccrj.2026.100221 (PMC13634569; doi:10.1016/j.ccrj.2026.100221)
Supplement: Supplementary file 1 [file mmc2.pdf]

FOOTPRINTS Scoping Survey

FOOTPRINTS Scoping Survey

Current PICU Bereavement Practices in Australia

We would like to invite you to participate in this survey exploring current bereavement practices in Australian Paediatric Intensive Care Units (PICUs).

The target population for this survey is one nursing representative per Australian PICU. We are inviting ANZICS Research Coordinators to complete this survey as nursing representatives for each site.

This survey forms part of phase one of the FOOTPRINTS bereavement project, which aims to develop and test a PICU bereavement service to follow-up families of children who have died an unexpected death.

If you choose to participate, the information that you provide in this survey will help us to obtain a baseline understanding of the contemporary clinical environment of current bereavement care practices in Australian PICUs.

This understanding will inform the development of an evidence-based service design for testing in phase two of the FOOTPRINTS project.

The FOOTPRINTS Project is a PhD project led by Arielle Jolly, CAHS - Telethon Nursing Research Fellow, Child and Adolescent Service and Curtin University, Western Australia, and supervised by Professor Fenella Gill, Dr Simon Erickson, Dr Kylie Davies, Dr Ashleigh Butler, and A/Prof Kristen Gibbons.

Your participation in this survey is completely voluntary. Completion of the survey should take no longer than 15 minutes, however there may be questions that you do not know the answer to. Please consult bereavement stakeholders in your organisation to gather the information required to answer all the questions. This may take additional time.

If you do not wish to participate, simply close your web browser.

If you consent to participate, please continue on and complete the survey questions. You can save your answers as you go through the survey. Once you have completed the survey, please click on the 'Submit' button.

Receiving your completed survey will indicate to the study team that you consent to participate, and to have the data that you provide included in this research project, as per the ethical approval granted for this project by the Child and Adolescent Health Service Human Research Ethics Committee.

If you have any questions about the ethical approval of this project, please contact CAHS.Ethics@health.wa.gov.au

If you have any questions about this research, please contact the Principal Investigator Arielle.Jolly@health.wa.gov.au

This survey asks questions about bereavement. If you experience any emotional distress while completing this survey, please contact:

- Your General Practitioner
- Life Line: 13 11 14
- Men's Line: 1300 78 99 78
- Beyond Blue: 1300 22 4636

Thank you for considering taking part in this study,

The FOOTPRINTS Project Team

PICU Details

What type of hospital is your PICU located in?

◯ Public
◯ Private
◯ Combined public and private

My PICU is

◯ Metropolitan
◯ Regional

Please identify the name of the hospital/PICU that your responses relate to. __________________________

What types of patients are admitted to your PICU?
You may select as many options as are relevant to your unit

☐ General paediatrics
☐ Cardiothoracic / cardiology
☐ Neurology / neurosurgery
☐ Other specialty - please state

Other Specialty __________________________

Role Details

What is your role?
You may select as many responses as are relevant to you

☐ Nurse Unit Manager
☐ Clinical Nurse Consultant
☐ Clinical Nurse Specialist
☐ Nurse Educator
☐ Research Coordinator
☐ Registered Nurse
☐ Other - please state

Other role __________________________

What is your highest level of education?

◯ Vocational training
◯ Diploma
◯ Degree
◯ Post Graduate Certificate
◯ Post Graduate Diploma
◯ Masters
◯ PhD

How many years of clinical experience do you have?

◯ 0 - 4 years
◯ 5 - 9 years
◯ 10 - 15 years
◯ 16 - 20 years
◯ 21 years and over

How many years of experience do you have working in PICU?

◯ 0 - 4 years
◯ 5 - 9 years
◯ 10 - 15 years
◯ 16 - 20 years
◯ 21 years and over

Model of Bereavement Services

What elements of bereavement care are currently being offered to families by staff in your PICU?

☐ Memory making activities - please describe
☐ Involvement of siblings in end-of-life activities - please describe
☐ End-of-life photography
☐ Family involvement in bathing or dressing the deceased
☐ Viewing the deceased in PICU
☐ Viewing of the deceased in the hospital mortuary
☐ Distribution of information to family members on hospital bereavement follow-up services
☐ Distribution of information to family members on community bereavement follow-up services
☐ Provision of sibling bereavement resources
☐ Sending of a card or e-mail of sympathy to bereaved families
☐ Other bereavement elements - please specify
☐ None

Please describe the memory-making activities in your PICU

Please describe how siblings are involved in end-of-life activities in your PICU

Other bereavement care elements offered

If a card or email of sympathy is sent, please describe:
a) How soon after the patient's death is it sent?
b) Who sends it?
c) To whom is it sent?

Does your PICU offer a bereavement follow-up service for families?
(A bereavement service is a systematic program of outreach care provided to bereaved families by your PICU)

◯ Yes - a formal, established service
◯ Yes - an informal service
◯ No
◯ I don't know

How long (in years) has your bereavement follow-up service been established?
Please state (if you don't know, state 'I don't know')

____

As part of the bereavement follow-up service is a telephone call to families offered?

◯ Yes
◯ No
◯ I don't know

Please indicate
a) How soon after the patient's death the call is made
b) The purpose of the call
c) Who makes the call

As part of the bereavement follow-up service is a visit to the PICU offered to families?

◯ Yes
◯ No
◯ I don't know

Please indicate:
a) How soon after the patient's death the visit is offered
b) How the visit is organised
c) What happens during the visit

Does your bereavement service include?

☐ Follow-up by a pastoral carer or religious representative
☐ Follow-up by a psychologist or counsellor
☐ Follow-up by physiotherapist or occupational therapist
☐ Follow-up by social worker
☐ Other, please specify

Other follow-up offered by

____

As part of the bereavement follow-up service is formal counselling to families offered?

◯ Yes
◯ No
◯ I don't know

Please state
a) Who offers the counselling?
b) When is it offered?
c) Where is the counselling held?

Is a meeting with PICU medical staff offered to the family as part of the bereavement follow-up service?

◯ Yes, routinely
◯ Yes, on request
◯ No
◯ I don't know

Please state
a) When is the meeting offered?
b) Where is it held?

As part of the bereavement follow-up service, are any other services or support offered to families?

◯ Yes - please state
◯ No
◯ I don't know

Other bereavement support

Workforce Model

How many staff are involved in the PICU follow-up bereavement service?

◯ One person
◯ 2 - 3 staff members
◯ 4 - 6 staff members
◯ In excess of 6 staff members
◯ I don't know

How many hours per week are allocated to the PICU bereavement follow-up service?
Please state (If you don't know, state 'I don't know')

____

Which member of staff is responsible for coordinating bereavement services in your PICU?

☐ Nurse Unit Manager
☐ Medical Officer
☐ Allied Health Professional
☐ Social Worker
☐ Clinical Nurse Consultant
☐ Clinical Nurse Specialist
☐ Nurse Educator
☐ Research Coordinator
☐ Registered Nurse
☐ Administrative Staff Member
☐ Other - please state
☐ I don't know

Please provide details of the staff member responsible for coordinating bereavement services in your PICU

Are PICU staff in the bereavement team?

◯ Permanent in the bereavement team role
◯ Rotating through the bereavement team role
◯ Mixture of permanent and rotating staff
◯ Other - please state
◯ N/A - our bereavement is delivered by non-PICU staff

Please provide detail of how staff are allocated to the bereavement team and the frequency of staff rotation

Are any staff given extra support or training for bereavement work?

◯ Yes - please state
◯ No
◯ I don't know

Please provide details of extra training or support provided to bereavement staff

Are staff offered debriefing after the unexpected or traumatic death of a patient?

◯ Yes, routinely
◯ Yes, on request
◯ No
◯ I don't know

Which form do debriefs take?

◯ Hot debriefs - held immediately after the death with staff present at the time
◯ Cold debriefs - held after the time of death with a structured multidisciplinary approach
◯ Both hot and cold debriefs
◯ Other - please state

Please describe how debriefs are facilitated

Does your bereavement follow up service receive dedicated funding?

◯ Yes - staff are given dedicated paid time to participate in the service
◯ Yes - staff are paid to participate, but are not allocated dedicated time in their workload for bereavement
◯ No - it is a voluntary service run by staff in addition to their usual role
◯ I don't know

Does your PICU offer routine PICU follow-up of patients who survive their admission?

◯ Yes - please describe
◯ Sometimes - please describe
◯ No
◯ I don't know

Please describe the PICU follow-up that currently occurs

Evaluation Model

Has the PICU bereavement service been evaluated?

◯ Yes
◯ No
◯ I don't know

How has the PICU bereavement follow-up service been evaluated?

☐ Verbal feedback from staff
☐ Verbal feedback from relatives
☐ Survey of staff
☐ Survey of relatives
☐ Interviews with staff
☐ Interviews with relatives
☐ Other - please state
☐ I don't know

Please describe the method of evaluation

How often does evaluation of the PICU bereavement follow-up service occur?

◯ Ongoing
◯ Yearly
◯ Other - please state
◯ I don't know

Frequency of evaluation

____

Have any practice changes been instituted in response to the evaluation of the PICU bereavement follow-up service?

◯ Yes - please describe
◯ No
◯ I don't know

Please describe changes made

What part of the PICU bereavement follow-up service appears to be most appreciated by families?

What part of the ICU bereavement follow-up service appears to be least liked by families?

What feedback do you receive from the PICU staff about the PICU bereavement follow-up service?

Are you considering implementing additional elements to PICU bereavement follow-up services?

◯ Yes - please state
◯ No
◯ I don't know

Please describe the additional bereavement elements planned

Do you have any further comments about bereavement follow-up services in PICU?

Do you have any information about your bereavement practices that you would like to share, such as guidelines or resources?

If so, please upload here.
