# Supplementary material for: Bereavement care practices: A national survey of Australian paediatric intensive care units
Source: Crit Care Resusc. 2026 Sep 23;28(4):100221. doi: 10.1016/j.ccrj.2026.100221 (PMC13634569; doi:10.1016/j.ccrj.2026.100221)
Supplement: Supplementary file 2 [file mmc1.docx]

Checklist for Reporting Results of Internet E-Surveys

| Checklist Item | Explanation | Page Number |
| --- | --- | --- |
| Describe survey design | Describe target population, sample frame. Is the sample a convenience sample? (In ‘open’ surveys this is most likely) | 3 – 4. |
| IRB approval | Mention whether the study has been approved by an IRB. | 5. |
| Informed consent | Describe the informed consent process. Where were the participants told the length of time of the survey, which data were stored and where and for how long, who the investigator was, and the purpose of the study? | 4.  Supp. Material |
| Data protection | If any personal information was collected or stored, describe what mechanisms were used to prevent unauthorised access. | 4. |
| Development and testing | State how the survey was developed, including whether the usability and technical functionality of the electronic questionnaire had been tested before fielding the questionnaire. | 3 – 4. |
| Open survey versus closed survey | An ‘open’ survey is open for each visitor of the site, while a closed survey is only open to a sample which the investigator knows (password-protected survey). | 4. |
| Contact mode | Indicate whether or not the initial contact with the potential participants was made on the internet. (Investigators may also send out questionnaires by mail and allow for Web-based data entry). | 4. |
| Advertising the survey | How/where was the survey announced or advertised? Some examples are offline media (newspapers), or online (mailing lists – if yes, which ones?) or banner ads (where were these banner ads posted and what did they look like?). It is important to know the wording of the announcement as it will heavily influence who chooses to participate. Ideally the survey announcement should be published as an appendix. | Supp. Material |
| Web/E-mail | State the type of e-survey (e.g. one posted on a website, or one sent out through email). If it is an email survey, were the responses entered manually into a database, or was there an automatic method for obtaining responses? | 3 – 4. |
| Context | Describe the website (or mailing list/newsgroup) in which the survey was posted. What is the website about, who is visiting it, what are visitors normally looking for? Discuss to what degree the content of the website could pre-select the sample or influence the results. For example, a survey about vaccination on an anti-immunisation website will have different results from a survey conducted on a government website. | 4 – 5. |
| Mandatory/voluntary | Was is a mandatory survey to be filled in by every visitor who wanted to enter the website, or was it a voluntary survey? | 4. |
| Incentives | Were any incentives offered? (e.g. Monetary, prizes, or non-monetary incentives such as an offer to provide the survey results)? | NA |
| Time/date | In what timeframe were the data collected? | 3. |
| Randomisation of items or questionnaires | To prevent biases items can be randomised or alternated. | NA |
| Adaptive questioning | Use of adaptive questioning (certain items, or only conditionally displayed based on responses to other items) to reduce number and complexity of questions. | Supp. Material |
| Number of items | What was the number of questionnaire items per page? The number of items is an important factor for the completion rate. | 4. |
| Number of screens (pages) | Over how many pages was the questionnaire distributed? The number of items is an important factor for the completion rate. | 4. |
| Completeness check | It is technically possible to do consistency or completeness checks before the questionnaire is submitted. Was this done, and if so, how? (usually JAVAscript)? An alternative is to check for completeness after the survey has been submitted (and highlight mandatory items). If this has been done it should be reported. All items should provide a non-response option such as ‘not applicable’ or ‘rather not say’ and selection of one option should be enforced. | 5.  Supp. Material |
| Review step | State whether respondents were able to review and change their answers? (e.g. through a back button or a review step which displays a summary of responses and asks the respondents if they are correct). | 4. |
| Unique site visitor | If you provide view rates or participation rates, you need to define how you determined a unique visitor. There are different techniques available, based on IP addresses or cookies or both. | 5. |
| View rate (Ratio of unique survey visitors/unique site visitors) | Requires counting unique visitors to the first page of the survey, dividing by the number of unique site visitors (not page views). It is not unusual to have view rates of less than 0.1% if the survey is voluntary. | Not done. |
| Participation rate (Ratio of unique visitors who agreed to participate/unique first survey page visitors) | Count the unique number of people who filled out the first survey page (or agreed to participate, for example by checking a checkbox), divided by visitors who visit the first page of the survey (or informed consent page, if present). This can also be called ‘recruitment’ rate. | 5. |
| Completion rate (Ratio of users who finished the survey/users who agreed to participate) | The number of people submitting the last questionnaire page, divided by the number of people who agreed to participate (or submitted the first survey page). This is only relevant if there is a separate informed consent page or if the survey goes over several pages. This is a measure for attrition. Note that ‘completion’ can involve leaving the questionnaire items blank. This is not a measure for how completely questionnaires were filled in. (If you need a measure for this, use the word ‘completeness rate’) | 5. |
| Cookies used | Indicate whether cookies were used to assign a unique user identifier to each client computer. If so, mention the page on which the cookie was set and read, and how long the cookie was valid. Were duplicate entries avoided by preventing users access to the survey twice; or were duplicate database entries having the same IP address within a given period of time eliminated before analysis? | Not done.  5. |
| IP check | Indicate whether the IP address of the client computer was used to identify potential duplicate entries from the same user. If so, mention the period of time for which no two entries from the same IP address were allowed (e.g. 24 hours). Were duplicated entries avoided by preventing users with the same IP access to the survey twice, or were duplicate database entries having the same IP address within a given period of time eliminated before analysis? | Not done.  5. |
| Log file analysis | Indicate whether other techniques to analyse the log file for identification of multiple entries. | Not done. |
| Registration | In ‘closed’ (non-open) surveys, users need to log in first and it is easier to prevent duplicate entries from the same user. Describe how this was done. | Not done. |
| Handling of incomplete questionnaires | Were only complete surveys analysed? Were questionnaires which terminated early (where, for example, users did not go through all questionnaire pages) also analysed. | Not analysed.  5. |
| Questionnaires submitted with an atypical time stamp | Some investigators may measure the people needed to fill in a questionnaire and exclude questionnaires that were submitted to soon. Specify the timeframe that was used as a cut off point and describe how this point was determined. | Not done.  5. |
| Statistical correction | Indicate whether any methods such as weighting of items or propensity scores have been used to adjust for the non-representative sample. | NA |
